# Current formula for calculating body mass index is applicable to Asian populations

**DOI:** 10.1038/s41387-018-0070-9

**Published:** 2019-01-28

**Authors:** Anoop Misra, Nikhil V. Dhurandhar

**Affiliations:** 1 Fortis-C-DOC Centre of Excellence for Diabetes, Metabolic Diseases and Endocrinology, New Delhi, India; 2 Department of Nutritional Sciences, Texas Tech University, Lubbock, TX 79409 USA

Body mass index (BMI, ratio of height and weight, expressed as kg/m^2^) is widely used to define overweight and obesity across many countries, populations, races and ethnicities. It is obvious that weight and height are linked. Hence, to adjust for the proportion between height and weight, the use of BMI assumes that in a given population, weight scales to height squared. This assumption, as well as the thresholds for defining overweight (BMI between 25 to <30 kg/m^2^) or obesity (BMI ≥ 30 kg/m^2^) have been derived from studying Caucasian population, hence may or may not apply to various other groups globally. In this issue of the journal, a team of researchers^1^ including obesity researchers, mathematicians and anthropologist asked following three questions: “(1) Does weight scale to height squared in Asian Indians? (2) Does the weight-height relationship differ within different Asian populations (i.e. between Asian Indians and South Koreans)? and (3) Do BMI thresholds for overweight and obesity for South Koreans differ from those for Caucasians?”

Hood et al.^1^ used a dataset of 43,880 adult Asian Indian males of age 15–54 y, including 5549 members of various tribes to conclude that weight does scale close to 2 (squared) in this geographically, socio-economically, culturally and ethnically diverse population. Even the tribal population, known for its smaller body size and stature compared to general population, showed similar scaling. Hood et al.^1^ concluded that BMI, as defined, does normalise weight for height in Asian Indians. Hence, it would be appropriate to use current formula for calculation of BMI in Asian Indian population. Next, they used the Korean National Health and Nutrition Examination Survey (KNHANES) data and observed an overlap between South Korean and Asian Indian population for weight and height. But also, there was a segment of Asian Indian population that was smaller in weight as well as height compared to South Korean population. Finally, using available percent body fat cut-offs and cardiometabolic risk factors, Hood et al.^1^ calculated BMI thresholds for South Korean males and females, respectively, as 22 and 18 kg/m^2^ for overweight and 26 and 23 kg/m^2^ for obesity. These thresholds are lower than the current BMI cut-offs applicable to Caucasian populations.

As outlined by the authors, the limitations of this study^1^ include the lack of data for women in Asian Indian dataset, so the applicability of BMI for Asian Indian women could not be tested. Unlike the South Korean dataset, the lack of body fat percent or cardiometabolic risk factors in the Asian Indian dataset prevented derivation of BMI thresholds for overweight and obesity in Asian Indian population. Nonetheless, similar conclusions were reached for Indians by Dudeja et al.^2^, following which a Consensus statement for Asian Indians suggested the BMI cut-offs for overweight as ≥23 kg/m^2^ and for obesity as ≥25 kg/m^2^ ^3^. These BMI cut-offs have been adapted by National Institute for Health and Care Excellence (UK) for migrant South Asians in UK as well^4^

The study by Hood et al.^1^ is important as it validates the use of BMI to study at least the Asian Indian and South Korean populations. Moreover, the study provides confidence to further research a very important health issue in South East Asian population. Obesity-related diseases, primarily type 2 diabetes in India and South Korea pose grim picture; in 2016, 4.8 million 30 y or older South Koreans (prevalence 13.7%) had diabetes, and about a quarter of population had prediabetes^5^. In 2017, 72.9 million Asian Indians in India (prevalence 8.8%) had diabetes and 24 million had  prediabetes^6^. Rising obesity seems to be a main contributor; and dysmetabolic state is mainly linked with excess body fat and ectopic fat deposition in various organs^7^. At a given BMI, the amount of total body fat and ectopic fat is different between Caucasians and Asians^8,9^. In such situations where excess body fat predominates but body weight is not very high relative to Caucasians, BMI may fail to classify obesity or may underestimate it^2,10^. In a sample of 1513 Hong Kong Chinese men and women, the risk of having diabetes mellitus or hypertension was at lower BMI or waist circumference levels in Chinese than in Caucasians^11^. In their analysis, Hood et al.^1^, reach similar conclusions in South Korean populations, using body fat as comparator.

Asians, and South Asians in particular, have more severe inflammation, insulin resistance, and liver fat even when non-obese by BMI standards used for Caucasians^9,12^. Excess liver fat may occur in non-obese south Asians as well, and has been shown to be almost double the  amount recorded  in Caucasians when matched for weight; and is associated strongly with insulin resistance^9,13^. Other adipose tissue depots such as the deep subcutaneous abdominal adipose tissue, which could destabilise metabolism, are more in Asian Indians than white Caucasians^14^. Specifically, adipocytes in deep subcutaneous abdominal adipose tissue are smaller and express more pro-inflammatory genes than adipose tissue from other subcutaneous sites, and its higher metabolic activity contributes to systemic insulin resistance^15^. In general, it appears that instead of overall increase in body fat, excess regional fat deposition (abdominal, intra-abdominal visceral, subcutaneous and specifically deep subcutaneous) builds up inflammatory and insulin resistant milieu in Asian Indians, which may contribute to the development of dysglycemia^16^.

Asians, particularly South Asians have lower muscle mass (and sarcopenia) compared to Caucasians^17^. When body fat is high and skeletal muscle is less, overall BMI may not be raised substantially. In such situation, do changes in both body compartments contribute to insulin resistance and diabetes? This question has not been answered with reasonable certainty. But some recent publications suggest that skeletal muscle mass as important as adiposity, if not more. Using baseline data from UK Biobank Study (n, 418,656), South Asians had ~5–6 kg lower handgrip strength (as a surrogate of sarcopenia) than white Europeans and Blacks and that lower handgrip strength was associated with higher prevalence of diabetes, independent of confounding factors. Importantly, attributable risk for diabetes associated with low handgrip strength was substantially higher in South-Asian than other ethnic groups in UK^18^.

Given these data, and in light of paper by Hood et al.^1^, is it time to dichotomise classification of obesity by BMI to at least two categories; existing cut-offs for Caucasians, and different, lower cut-offs for Asian population? Asian population should probably have interventions at lower BMI to prevent diabetes and related complications^10^. Changes in guidelines would mean an increase in the number of individuals considered to have overweight or obesity. Consequentially, upgradation of health resources to tackle this additional burden will be required in many Asian countries. But this awareness could be an important step for containment of diabetes epidemic in Asian countries.
